# Laser Capture Microdissection in Dentistry

**DOI:** 10.1155/2010/592694

**Published:** 2011-01-13

**Authors:** Uraiwan Chokechanachaisakul, Tomoatsu Kaneko, Takashi Okiji, Reika Kaneko, Hideaki Suda, Jacques E. Nör

**Affiliations:** ^1^Pulp Biology and Endodontics, Department of Restorative Sciences, Graduate School, Tokyo Medical and Dental University, 5-45, Yushima 1-chome, Bunkyo-ku, Tokyo 113-8549, Japan; ^2^Division of Cariology, Operative Dentistry and Endodontics, Department of Oral Health Science, Graduate School of Medical and Dental Sciences, Niigata University, 2-5274, Gakkocho-dori, Chuo-ku, Niigata 951-8514, Japan; ^3^Department of Applied Molecular Medicine, Niigata University Graduate School of Medical and Dental Sciences, Niigata 951-8510, Japan; ^4^Department of Cariology, Restorative Sciences, and Endodontics, Dental School, University of Michigan, 1011 N. University, Ann Arbor, MI 48109-1078, USA

## Abstract

Laser capture microdissection (LCM) allows for the microscopic procurement of specific cell types from tissue sections that can then be used for gene expression analysis. According to the recent development of the LCM technologies and methodologies, the LCM has been used in various kinds of tissue specimens in dental research. For example, the real-time polymerase-chain reaction (PCR) can be performed from the formaldehyde-fixed, paraffin-embedded, and immunostained sections. Thus, the advance of immuno-LCM method allows us to improve the validity of molecular biological analysis and to get more accurate diagnosis in pathological field in contrast to conventional LCM. This paper is focused on the presentation and discussion of the existing literature that covers the fields of RNA analysis following LCM in dentistry.

## 1. Introduction

Several experimental techniques are available for molecular profiling studies such as DNA microarray, differential display, serial analysis of gene expression, massive parallel signature sequencing, and suppression subtractive hybridization [1]. Although useful, shortcomings with these systems are often encountered especially in input DNA, RNA, or proteins from pure population [2]. For example, surgical samples are variable in shape and size, and are often a mixture of several kinds of tissues. Thus, the outcome of molecular biological analyses from these samples may not be accurate. The laser capture microdissection- (LCM-) based molecular biological analysis has been developed as a powerful methodology that improves these problems [2–4]. 

**Figure 1:**
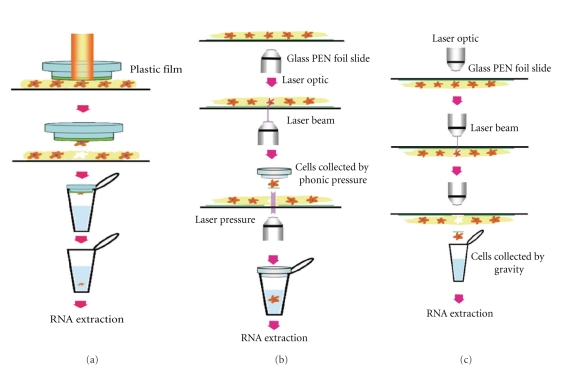
(a) Principle of the Arcturus laser capture microdissection. A plastic film is covered over the specimen. When the plastic film is removed, the dissected tissue by IR laser is attached to the film and isolated from the rest of the sample section. (b) Principle of the Zeiss's PALM microdissection. The tissue section has been mounted on a PEN foiled slide. Then, UV laser beam focused and cut a contour around the area of the target tissue via inverted microscope. The dissected tissue is collected by photonic pressure; using laser pressure to lift the dissected tissue into a collecting cap is named laser pressure catapulting. (c) Principle of the Leica AS LMD microdissection. The tissue section that has been mounted on a PEN foiled slide is set upside down of the stage. Then, laser beam dissect the target tissue. The dissected tissue falls into collecting cap positioned under the specimen.

LCM was first introduced as a system that is able to retrieve defined cell population from human tissue samples. The original system was invented by the National Institutes of Health [2] to isolate specific cells from histological slides under microscope. Nowadays a variety of LCM apparatus are available and their major differences relate to how they collect dissected cells. For example, the PixCell system (Arcturus, MDS Analytical Technology, CA, USA) uses both ultraviolet (UV) laser to cut and infrared (IR) laser to collect cells (Figure 1(a)). Zeiss's PALM system (a subsidiary of Carl Zeiss MicroImaging, Jana, Germany) uses UV laser to cut the tissues via inverted microscope and collect cells by photonic pressure (Figure 1(b)). Leica AS LMD system (Mannheim, Germany) uses a UV laser to cut, and then dissected cells fall into a collecting tube by gravity (Figure 1(c)). 

 Analyses using LCM technology have been further developed/improved and performed in various fields. In dentistry, this technology has been utilized in different research fields such as oral embryology [5–10], oral oncology [11–16], oral cell biology [7, 17–21], and tissue engineering including teeth [17, 22–24]. In this paper, we will focus on the presentation and discussion of existing literature that covers the dental researches using LCM, especially in the field of RNA analysis. 

## 2. Oral Cancer

Oral cancer is a type of head and neck cancers developed in any part of the oral cavity or oropharynx. When oral cancer spreads (metastasizes), it usually travels through the lymphatic system and appears first in nearby lymph nodes in the neck. The new tumor at the metastatic site has the same kind of abnormal cells as the primary tumor. Although the recently gained knowledge of normal and aberrant function of oncogenes and tumor suppressor genes has provided unique opportunities to understand, and ultimately to control the processes leading to malignancy, the molecular mechanisms of this disease remain poorly understood [15].

The recent development of hybridization-based methods utilizing cDNA arrays, provides an opportunity to identify genes expressed in normal and tumor tissues, as well as to analyze gene expression profiles in tumor progression. However, an accurate procurement of specific cell types for RNA isolation is a critical step influencing the validity of this analysis. On this point of view, LCM provides a great advantage since it enables the procurement of pure cell populations from tissue sections, a key consideration as many tumors are heterogeneous, and include areas of connective tissues, blood vessels, and even inflammatory cells that infiltrate into the tumor mass. The use of LCM to harvest cells from their native tissue environment, followed by the use of high-density oligonucleotide probe arrays to identify gene expression differences between normal and malignant oral epithelial cells, provide powerful means to decode the molecular events involved in the genesis and progression of oral cancer. 

In oral cancer tissues, a number of genes have been identified by either LCM/oligonucleotide microarray approach [15] or the LCM/cDNA library approach [14] to be highly expressed/upregulated. Leethanakul et al. [14, 15] used LCM and cDNA arrays, which approach allowed the detailed analysis of gene expression and provided the first evidence for the feasibility of performing a comprehensive molecular characterization of normal, premalignant, and malignant head and neck squamous cell carcinoma (HNSCC) cells. Biopsies from HNSCC patients were snap frozen and HNSCC cells were harvested from hematoxylin-stained section using PixCell LCM for studying differential gene expression and GAPDH mRNA. Results demonstrated that high-quality, representative cDNA libraries can be generated from microdissected OSCC tissue. In another study, Alevizos et al. [13] applied LCM to study differential gene expression from solid tumor tissues. The cancer tissues were snap frozen after surgical removal, and the PixCell system (LCM/GeneChip analysis) was successfully used to harvest normal and tumor cells for oligonucleotide microarray analysis and real-time PCR of collagenase, urokinase plasminogen activator, and cathepsin L. Results from these studies support the notion that analyzing LCM-derived RNA with microarrays provides powerful approaches for identifying candidate genes that have not been implicated in oral cancer.

LCM method can minimize possible contamination from infiltrated inflammatory cells so that cancer cells were differentially captured from the tumor tissue [11], cancer tissues were immediately fixed with formalin and embedded in paraffin. The sections were stained with hematoxylin and eosin and subjected to the PALM LCM to analyze IgG1, V3, and V4f mRNA. Using *in situ* hybridization and LCM-correlated RT-PCR, the authors found that IgG heavy chain transcripts were present in the oral cancer cells and in some normal epithelial cells adjacent to the tumor. These findings suggest that tumor-derived immunoglobulin may be an important new target molecule for tumor biotherapy. 

In clinical application, the use of LCM technologies therefore allows for scanning of gene expression patterns and search for those correlating with a disease state. Normally, clinical staging of cervical lymph nodes is carried out by clinical examination of the neck region or by ultrasound, computed tomography, and magnetic resonance imaging. However, the sensitivity of these methods is still limited due to the fact that the false negative rate is high in clinically diagnosed metastasis-negative (N0) patients. In an effort to find new biomarkers that will provide more accurate diagnosis and safer and more efficacious treatment for oral squamous cell carcinoma (OSCC), Nguyen et al. applied LCM and microarray technology to investigate the differences in gene expression profiles between primary OSCC metastasized and no metastasized to cervical lymph nodes [25]. Primary oral specimens were embedded with OCT and frozen sections were fixed with cold ethanol. OSCC cells were obtained accurately from the hematoxylin-stained tissue sections by LCM. Then they successfully selected genes that showed a difference in expression levels between the two groups, using DNA-Chip analysis software for high-level analysis [25]. 

**Figure 2:**
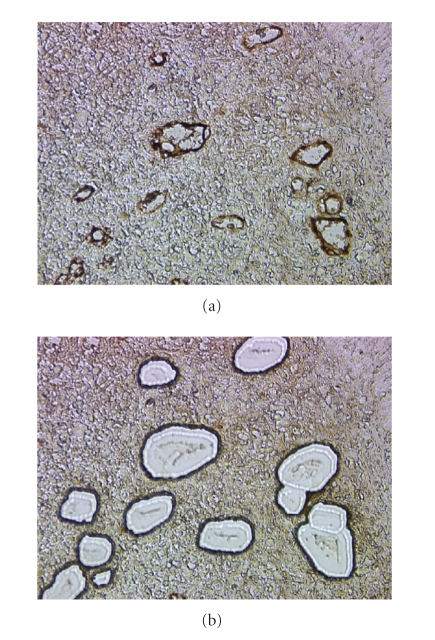
Immune-LCM used for retrieval of Factor VIII-positive endothelial cells from FFPE tissue sections. (a) Factor VIII+ endothelial cells in the head and neck carcinoma. (b) Retrieval of Factor VIII-positive endothelial cells. This step is performed after dissecting blood cells in Factor VIII-positive capillaries.

We have recently developed a methodology of immune-LCM of formaldehyde-fixed, paraffin-embedded, and coagulation factor VIII-immunostained head and neck carcinoma by using Leica LMD system [15]. This method allows us to correct RNA of factor VIII-positive endothelial cells retrieved from the tumor mass (Figure 2). This method may be ideally suited for the analysis of relatively rare cell types within a tissue and should improve on our ability to perform differential diagnosis of pathologies as compared to conventional LCM [3].

## 3. Tooth Development

Tooth development is the cumulative result of signaling of growth factors. Initial molecular signals in the dental epithelium induce gene expression in the adjacent dental mesenchyme. Reciprocal signaling between the epithelial and mesenchymal tissues continues throughout tooth development, resulting in the formation of a tooth of specific size and shape. 

Werner et al. studied the role of macrophage colony stimulating factor (CSF-1) isoforms on tooth morphogenesis by using PixCell II LCM to capture ameloblasts and odontoblast from HistoGene LCM-stained frozen tissue sections, followed by the mRNA expression analysis using RT-PCR. The results showed that CSF-1 is important for optimal cytodifferentiation and dentin matrix protein-1 (DMP-1) expression [6]. Absence of CSF-1 receptor was associated with shortened ameloblasts, loss of odontoblastic polarization, and dramatically reduced DMP-1 expression. csCSF-1 may act in an autocrine fashion in odontoblasts to regulate DMP-1 since odontoblasts express the CSF-1R and may be involved in mediating tooth matrix formation [6, 10]. 

### 3.1. Tooth Eruption

Tooth eruption requires alveolar bone resorption as well as possible bone growth at the base of the tooth. The detailed mechanisms of tooth eruption is yet to be clarified, mainly because many issues regarding growth factors, such as types of expressing cells, temporo-spacial changes of expression patterns, and results of growth factor interaction, remain fully understood, LCM is applicable to molecular profiling of tissue specimens, permitting correlation of cellular and molecular signatures with specific cell population, especially comparison of cellular elements within the tissue microenvironment or in the variety of cell types. Thus, LCM has been applied to the studies of tooth eruption including evaluation of the tissue surrounding unerupted tooth, dental follicle microenvironment, factors involved in tooth eruption process, time expression of growth factors, and/or cytokines and type of cells releasing growth factors to conclude mechanism of tooth eruption [5, 8–10, 26].

The use of LCM enables collecting interested and specific cells such as stellate reticulum (SR) and dental follicle cells from the tissue surrounding unerupted tooth [5, 8]. LCM has also been used for study of time releasing factors for tooth eruption that cells retrieved from different age of tissues [10, 26]. In these studies, PALM LCM was used to purify SR cells collected from liquid nitrogen-frozen mandible and gene expression of factors involved in tooth eruption, such as parathyroid hormone-related protein (PTHrP) and in vitro effect of PTHrP on gene expression of VEGF and BMP-2, was analyzed. After collecting cells, the RT-PCR and/or cDNA methodologies were used. Result showed that PTHrP was maximally expressed at day 7 postnatally in the SR and still high at day 9 [5].

Wise and Yao studied gene expression of bone morphogenetic protein-2 (BMP-2) and receptor activator of nuclear factor kappa B ligand (RANKL), as a marker gene for alveolar bone formation, and resorption, respectively, in the dental follicle harvested by liquid nitrogen frozen mandible of new born rats, using PALM LCM [8]. The authors found that RANKL showed a higher expression in the coronal half of the follicle, whereas BMP-2 showed a higher expression in the basal half [8]. 

The regional differences in the expression of the bone-related genes are not the sole factors regulating bone resorption/formation during tooth eruption. Thus, cytokines and growth factors involved in bone resorption process were studied on their influence on the tooth eruption. IL-10 is an anti-inflammatory cytokine that inhibits osteoclast formation and may act to suppress the alveolar bone resorption. Liu et al. studied the dental follicle tissue harvested from cold ethanol-fixed, toluidine blue-stained sections of liquid nitrogen frozen-mandibles by using PALM LCM [9]. They found that IL-10 and IL-10R were expressed in the dental follicle of postnatal rats by RT-PCR [9]. Expression of PTHrP gene was also found in the stellate reticulum adjacent to the dental follicle, using similar strategy [10].

### 3.2. Periodontal Ligament

The periodontal ligament (PDL) is classified as connective tissue, but its histochemical [18] and genetic [27] properties are different from other connective tissues. Mature PDL is very active in reabsorbing and secreting collagen to maintain PDL fibers under stress of mastication. Many investigations have been performed on the PDL to identify the molecular biological characteristics differing from other connective tissues [28–30]. Analyzing the PDL tissue attached to a root following tooth extraction is subjected directly to molecular biological analysis for *in vivo* studies [31], and cultured tissues and outgrown cells are used for *in vitro* studies [27, 32]. However, there is difficulty to obtain the target tissues and/or cells without contaminations of other tissues and/or cells *in vivo*. On the other hand, *in vitro,* the environment of the cells may be changed during cell culture and thus may influence the expression of molecular characteristics from original tissues. Thus, it would be more reliable to extract the sample directly from the tissues by using LCM.

Nakamura et al. retrieved the PDL directly from toluidine blue-stained sections of liquid nitrogen-frozen rat molars by using PALM LCM and examined the gene expression of transforming growth factor (TGF)-*β*1, which plays an important role in the modulation of tissue formation and development of the PDL, to compare with the pulp and subgingival connective tissues [18]. The authors successfully compared the gene expression levels among the three tissues directly from undecalcified frozen sections [18]. 

LCM, however, has the limitation that it is difficult to microdissect the cells located near hard tissues, such as osteoblasts, odontoblasts, and cementoblasts, and also mineralized tissues, even at the maximum output of the laser beam. Reducing the thickness of the section would be necessary for this purpose [18].

Diseased periodontal tissues have also been studied using LCM. Three major events are associated with the initiation and progression of periodontal attachment loss; local epithelial cell proliferation and migration, dissolution of Sharpey's fibers with loss of attachment, and ultimately resorption of alveolar bone. However, regulation of these processes is poorly understood, although local expression of cytokines and growth factors likely play significant roles. In one study, the Arcturus LCM technology was successfully used to search for the source of gene expression of KGF, KGFR, K19, and type I collagen, by dissecting target cells from snap-frozen, acetone-fixed sections of pooled control and diseased tissues followed by RT-PCR amplification [20]. We recently performed LCM for formaldehyde-fixed, demineralized, and frozen sections of rat PDL, and retrieved the inflamed or normal tissues from furcal PDL by using the Leica LMD system [33]. This research allowed us to quantify mRNAs of some antigen presenting cell-related molecules such as CD86 and CD83.

## 4. Pulp Biology

The dental pulp is the part in the center of a tooth made up of living soft tissue, and odontoblasts contain large nerve trunks and blood vessels to functions of dentin formation, nutrition, sensory and protection.

Dental pulp may be capable of regenerating following injury, but the specific mechanisms underlying pulp regeneration and reparative dentinogenesis have still to be clarified. It is due in part to the limitation to obtain sufficient number of pure and intact cell population and maintain those cells in culture. Thus, cell differentiation and dentin formation processes, in relation to the genetic profile of specific types of cells, for example, odontoblasts, are not completely known. LCM technology may overcome most of these problems, since it allows pure population of defined cells to be isolated from frozen or paraffin tissue sections and RNA retrieval from these cells for studies of gene expression of, for example, growth factors.

Hoffmann et al. used PALM-LCM to retrieve homogeneous population of odontoblasts followed by RT-PCR analysis to determine the exact molecular mechanism leading to cell differentiation, function, and biochemical profile of these cells [23, 24]. Cryostat sections of heads from mouse embryo and pulps were processed for LCM. The laser was focused through the objective lens of an inverted microscope on a circumscribed area within the cusp tip. In the sections from embryos, the microbeam was directed to the peripheral zone of dental papilla cells. Laser pressure catapulting was used to eject selected clusters of odontoblasts into a microfuge cap. Total RNA was measured by RT-PCR analysis with probes of dentin matrix genes, *α*1 collagen, dentin sialophosphoprotein (DSPP), and osteocalcin. Paraffin-embedded sections were also prepared to perform in situ hybridization technique to detect the expression of these genes in vivo. These studies showed that progenitors and differentiated odontoblasts were easily identified under LCM. Therefore, LCM appeared a powerful tool to procure a homogeneous population of odontoblasts from tissue sections and allowed to retrieve a clear-cut odontoblast monolayer; target cells were easily distinguished by their morphology in unstained sections, but the stain was useful for identifying captured clusters of odontoblasts within the microfuge caps [23, 24].

Chronic bacterial infection of the root canal system causes the establishment of periapical inflammatory lesions as a result of the activation of local defense reactions against intracanal pathogens. Immunological responses also have critical roles to establish the lesions, because bacterial elements potentially activate various forms of immune responses by acting as antigens. We performed LCM for HLA-DR-positive dendritic cells and macrophages in human radicular granulomas by using the Leica LMD system. We demonstrated that, in the lymphocyte-rich area, expression levels of HLA-DR alpha-chain, CD83, and CD86 mRNAs were higher in HLA-DR-positive dendritic cell as compared with HLA-DR-positive macrophages. The result suggests that dendritic cells in radicular granulomas act as stronger antigen-presenting cells, as compared with macrophages [21].

## 5. Tissue Engineering

Tissue engineering/regenerative medicine is an emerging multidisciplinary field involving biology, medicine, and engineering that is likely to revolutionize the ways improving the health and quality of life by restoring, maintaining, or enhancing tissue and organ function. 

LCM is a new technology that overcomes the problem of inaccurate results due to contaminated tissues. The study of gene expression profiles is a pivotal instrument for development biology. For the investigation and analysis of molecular interaction, in situ hybridization, immunohistochemical localization, and histomorphological studies have been the methods of choice to study the molecules of interest in the tissues. 

Young et al. demonstrated the successful bioengineering of complex tooth crowns closely resembling those of naturally developing teeth [22]. To confirm the identity of putative odontoblast-like cells present in bioengineered tooth tissues, Leica LCM was used to perform RT-PCR analyses from paraffin-embedded sections. The results showed that DSPP product was generated from control porcine third molar and tissue-engineered odontoblasts. The analysis of morphology, histology, immunohistochemistry, and LCM RT-PCR analysis demonstrated the successful engineering of recognizable tooth structures exhibiting the cellular organization and presence of appropriate proteins found in natural teeth such as a morphologically correct enamel organ consisting of stellate reticulum, stratum intermedium, ameloblasts, and dental enamel. In addition, putative Hertwig's root sheath epithelia were also present [22].

**Figure 3:**
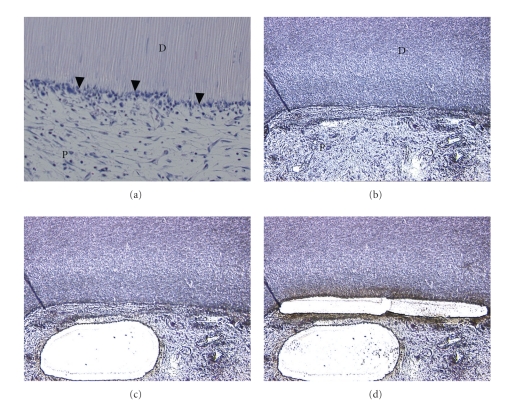
Step-by-step characterization of the technique based on LCM used for retrieval of either the odontoblastic layer or the tissue underneath the odontoblastic layer from formaldehyde-fixated paraffin embedded tissue sections that had been demineralized. (a) H&E staining of the engineered dental pulp-like tissue after 21 days of implantation. D: dentin. P: engineered dental pulp. Arrowheads: odontoblastic layer. (b) Air dried slide of the engineered dental pulp-like tissue. D: dentin. P: engineered dental pulp. (c) Removal of the tissue underneath the odontoblastic layer. (d) Retrieval of the odontoblastic layer.

We recently performed LCM for engineered pulp tissue samples using Leica LMD (Figure 3) [17]. The samples were fixed with formalin, demineralized, and embedded in paraffin. Notably, the samples allowed us to perform a gene expression analysis of DSPP by using real-time PCR [34]; we demonstrated the upregulation of DSPP mRNA in tissues of the odontoblast layer just underneath the dentin, as compared with the tissues underneath the odontoblast layer. This method constitutes a new approach for gene expression studies of mineralized tissues such as bone and teeth, and opens the door for the acquisition of new data from archived specimens.

## 6. Tissue Preparation of Hard Tissue Sample for Immune-LCM

Tissue preparation of hard tissue sample is important for immune-LCM, if the target tissue is surrounded by calcified tissues such as enamel/dentin and bone. In our research, we performed tissue fixation and demineralization as follows: fixation of the tissues with 10% neutral buffered formaldehyde at 4°C, and demineralization with 10% formic acid at 4°C. Paraffin embedding was performed as follows: dehydration in 70% ethanol for 30 minutes, 90% ethanol for 1 hour, 95% ethanol for 30 minutes at 4°C, and 3 times in 100% ethanol for 1 hour at room temperature, immersion 2 times in xylene for 1 hour at room temperature, 4 times in infiltrating paraffin for 30 minutes at 58°C, and embedding in paraffin. Sectioning of the paraffin embedded samples at 5 *μ*m thickness was performed on a microtome with a new sterile disposable blade. Sample sections were mounted on poly-L-lysine coated glass foiled polyethylene naphthalate (PEN) slides for LCM (Leica Microsystems, Bannock Burn, IL). The slides were dried in an incubator at 35°C for 6 hours. Nuclear staining by hematoxylin was performed as follows: deparaffination of the slides twice in xylene for 3 minutes at room temperature, washing 3 times in 100% ethanol for 30 seconds, 90% ethanol for 30 seconds, 70% ethanol for 1 minute, and in RNase-free water for 30 seconds at 4°C, immersion in hematoxylin for 5–10 seconds at room temperature, followed by washing with RNase-free water for 30 seconds at 4°C. The slides were let to dry at 4°C and keep in freezing compartment for preservation of RNA until immune staining [3].

## 7. Conclusion

Laser capture microdissection is a useful method for obtaining microscopic samples as small as individual cells from tissues for molecular analysis. The different outcome of the various LCM studies are likely reflective of the experimental approaches and methods of analyses. First, retrieving RNA by using LCM avoids contamination of heterogeneous cellular elements. Second, sample number and the type of gene expression analysis used may be relevant to the discrepancies. Third, the stage of the tissue, source, and anatomical site of the cells, and handling methods can further result in different gene expression levels.

 An advantage of the LCM approach is that only a small amount of starting material is required for the extraction of a sufficient quantity of total RNA. Furthermore, the quality and integrity of the RNA make this approach suitable for use with available array technology, thus affording the possibility to define a pattern of gene expression because the combination of using LCM and applied RT-PCR protocol allows the specific isolation and characterization of selected cells [35]. The use of LCM technologies therefore allows for the scanning of gene expression patterns and the search for those correlating with a disease state. Comparative analysis of gene expression profile may help identify aberrant expressed or mutate gene. Furthermore, gene expression profiles can now be investigated within histologically defined, homogeneous population of cells by using LCM, thus affording the possibility of these newly available techniques being applied to investigations of expression patterns in normal as well as in neoplastic tissues.
